# Monocyte counts are negatively associated with ankle-brachial index values in non-dialysis-dependent chronic kidney disease patients

**DOI:** 10.1080/0886022X.2020.1796704

**Published:** 2020-07-28

**Authors:** Myung Sung Kim, Dong-Jin Oh

**Affiliations:** Department of Internal Medicine, Myongji Hospital, Hanyang University College of Medicine, Goyang, Korea

**Keywords:** CKD, ankle-brachial index, leukocyte, subtypes, monocyte

## Abstract

Our aim was to determine which leukocyte subtypes are most relevant to ankle-brachial index (ABI) values in patients with non-dialysis-dependent chronic kidney disease (NDD-CKD). The study included 79 NDD-CKD patients aged 62.84 ± 12.09 years (63.33% men; 26.67% patients with diabetes) and 21 age-matched normal controls. According to the estimated glomerular filtration rate (eGFR) calculated by the CKD-Epidemiology Collaboration equation (CKD-EPI), we classified the study population into 2 groups (21 subjects with NDD-CKD with an eGFR 60–89 mL/min/1.73m^2^, 58 subjects with NDD-CKD with eGFR <60 mL/min/1.73 m^2^). ABI was calculated as the ratio of the ankle systolic BP divided by the arm systolic BP using an ABI-form device. An automated hematologic analyzer was used to measure total and differential leukocyte counts. Monocyte counts and monocyte-to-total leukocyte count ratios (MTR) in patients with an ABI value <1.10 were significantly higher than those in patients with an ABI value ≥1.10, respectively. Univariate analyses revealed that mean ABI values were negatively correlated with monocyte count (*r*= −0.341; *p* = 0.044), MTR (*r*= −0.346, *p* = 0.031). Multivariate linear regression analyses showed that monocyte count was negatively associated with ABI values (β ± SE = −1.825 ± 0.341, *p* = 0.013). The area under the curve of monocyte counts was 0.695 (95% confidence interval 0.586–0.804, *p* = 0.002) in predicting an ABI value <1.10. Monocyte counts are negatively associated with ABI values in patients with NDD-CKD without apparent peripheral arterial occlusive disorder (PAOD).

## Introduction

Chronic kidney disease (CKD) patients have high morbidity and mortality rates, mainly due to cardiovascular disease (CVD) events such as coronary arterial occlusive disorder (CAOD), cerebral vascular disorder and peripheral arterial occlusive disorder (PAOD) [1,2]. Cold extremities, peripheral numbness, and frequent muscle cramps are very commonly reported by patients with CKD; these symptoms can be caused by peripheral ischemia related to PAOD and main CVD events. Previous studies reported a high prevalence of PAOD in patients with CKD [3,4]; this problem is becoming more severe because of the increasing number of diabetic and elderly patients within the CKD and dialysis populations.

Atherosclerosis is a chronic inflammatory disease marked by early leukocyte recruitment that continues throughout plaque formation and rupture [5,6]. Total leukocyte count elevation was associated with impaired epicardial and myocardial perfusion and 1.4-fold risk of coronary heart disorder (CHD) [7,8]. Elevated total leukocyte count was also associated with PAOD [9,10].

There have been several epidemiologic studies about the associations between total leukocyte count/specific leukocyte subtype such as neutrophils, monocytes, neutrophil-to-lymphocyte ratio (NLR), lymphocyte-to-monocyte ratio (LMR), and carotid atherosclerosis, apparent PAOD including critical limb ischemia (CLI) in the general population and in patients with diabetes [11–13]. However, previous studies have targeted on the association between leukocyte subtype and patients with apparent PAOD, CLI, an ankle-brachial index (ABI) value <0.9 [11,14]. It is true that there have been no studies to investigate the relationship between ABI values and total/partial leukocyte counts or subtypes, especially in non-dialysis-dependent chronic kidney disease (NDD-CKD) patients without evidences of PAOD and CLI. Therefore, our aim was to determine which leukocyte subtypes were most relevant to ABI values in NDD-CKD patients with no evidences of PAOD and CLI.

## Patients and methods

### Study population

We enrolled 79 NDD-CKD patients aged 62.84 ± 12.09 years (63.33% men; 26.67% patients with diabetes, non-smoker) and 21 normal controls (age-matched healthy persons [63.91 ± 3.56 years] who had an estimated glomerular filtration rate (eGFR)>90 mL/min/1.73 m^2^ without pathological or radiologic or urine [proteinuria] or blood [electrolyte] diseases) who received medical checkups without a specific past history in our hospital. The cutoff value of mild renal function reduction is considered to be more than eGFR 60 mL/min/1.73m^2^ and the number of patients enrolled in our study is relatively small. Therefore, we classified the patient population into 2 groups (21 subjects with NDD-CKD having pathological [renal biopsy], urine [proteinuria], blood [electrolyte] or imaging abnormalities with an eGFR 60–89 mL/min/1.73 m^2^, 58 subjects with NDD-CKD with an eGFR < 60 mL/min/1.73 m^2^) by referring to a previously published paper [15]. eGFR was calculated by the CKD-Epidemiology Collaboration equation (CKD-EPI). We excluded patients with dialysis-dependent chronic kidney disease (DD-CKD), PAOD such as gangrenous changes in both foot, amputees, and patients undergoing percutaneous transluminal angioplasty (PTA) and critical limb ischemia (CLI), treatment of immunosuppressive agents including corticosteroid, active hematological pathology. CLI was defined by most vascular clinicians in patients who present with lower extremity ischemic rest pain, ulceration, or gangrene [16]. In these individuals, the untreated natural history of severe PAOD would lead to major limb amputation within 6 months. CLI was also a component of the Fontaine clinical stages 3–4 and the Rutherford clinical categories 4–6 [16]. And patients were classified into two groups based on a median value of ABI. The protocol was approved by our Institutional Review Board [MJH201601123021-HE001] and conducted in accordance with the Declaration of Helsinki. All enrolled patients gave written informed consent.

### Biochemical measures

Blood samples were obtained at routine outpatient clinic visits or medical checkups for stage 1–5 NDD-CKD patients and normal controls. Blood samples were analyzed for uric acid, phosphorus (P), calcium (Ca), and intact parathyroid hormone (PTH) concentrations using previously established methods with ranges of 3.0–7.0 mg/dL, 1.9–3.4 mg/dL, 8.2–10.4 mg/dL, and 12–72 pg/mL, respectively. In addition, high-sensitivity CRP (hs-CRP) and low-density lipoprotein (LDL) cholesterol was measured. Ethylene diamine tetra acetic acid (EDTA)–containing tubes are used for complete blood counts (CBC), and total and differential leukocyte counts including neutrophil, lymphocyte, monocyte, basophil, and eosinophil were determined using a Sysmex XN-20 (Sysmex Corporation, Kobe, Japan) within 1–2 h after venous puncture. Neutrophil-to-total leukocyte count ratio (NTR), lymphocyte-to-total leukocyte count ratio (LTR), monocyte-to-total leukocyte count ratio (MTR), NLR, LMR, neutrophil-to-monocyte ratio (NMR), and neutrophil-to-sum of lymphocyte and monocyte count ratio (NLMR) were calculated. Enrolled patients had the results of CBC more than three times a year prior to the ABI measurements except controls. Thus, the average value of three CBC tests was used in our study. BP was measured three consecutive times in a sitting position using a conventional sphygmomanometer by the same nurse on each occasion. The mean arterial pressure (MAP) was calculated using the following formula: [systolic blood pressure (SBP) +2 × diastolic blood pressure (DBP)]/3. Pulse pressure (PP) was calculated using the following formula: SBP-DBP.

### ABI measurement

ABI values were measured using an ABI-form device (VP1000; Colin Co. Ltd., Komaki, Japan), which automatically and simultaneously measures the blood pressure (BP) in both arms and ankles using an oscillometric method. ABI was calculated as the ratio of the ankle systolic BP (posterior tibial artery) divided by the arm systolic BP (brachial artery), and the lowest ankle systolic BP value was used in the calculation. And, we used the mean value of both limb measurements.

### Statistical analysis

Because of the small number of patients, normal distribution was tested using a single sample Kolmogorov–Smirnov analysis. Variables are expressed as mean ± standard deviation (SD). Between-group differences were assessed for significance using Mann–Whitney *U* tests. Spearman’s (non-parametric) correlations were used to test for associations between ABI and selected clinical, anthropometric, biochemical, and leukocyte subtypes. Multivariate linear regression analyses were performed to test the associations between ABI and selected clinical, anthropometric, biochemical, and leukocyte subtypes. We evaluated the receiver operating characteristics (ROC) curve of monocyte counts for predicting ABI in our study population. Statistical analyses were performed using SPSS software version 14.0 (SPSS Inc., Chicago, IL, USA). *p* Values <0.05 was considered statistically significant.

## Results

### Characteristics of NDD-CKD patients

Mean total leukocyte, neutrophil, lymphocyte, and monocyte counts (/mm^3^) were 6494.94 ± 1796.57, 3651.27 ± 1340.11, 2192.58 ± 770.71, and 535.70 ± 189.61, respectively. Mean NTR, LTR, MTR, NLR, NMR, LMR, and NLMR were 0.56 ± 0.09, 0.34 ± 0.09, 0.08 ± 0.02, 1.42 ± 3.10, 7.23 ± 2.50, 4.43 ± 1.76, and 1.47 ± 0.96, respectively. Other clinical and biochemical characteristics of NDD-CKD patients are shown in Table 1.

**Table 1.** Characteristics of NDD-CKD patients (*N* = 79).

Variable	Value
Age (years)	62.84 ± 12.09
Male sex (%)	63.33
DM (%)	26.67
HTN (%)	39.24
Creatinine (mg/dL)	1.90 ± 1.11
eGFR by CKD-EPI equation (mL/min/1.73m^2^)	38.15 ± 24.14
Total leukocyte count (/mm^3^)	6494.94 ± 1796.57
Neutrophil count (/mm^3^)	3651.27 ± 1340.11
Lymphocyte count (/mm^3^)	2192.58 ± 770.71
Monocyte count (/mm^3^)	535.70 ± 189.61
NTR	0.56 ± 0.09
LTR	0.34 ± 0.09
MTR	0.08 ± 0.02
NLR	1.42 ± 3.10
NMR	7.23 ± 2.50
LMR	4.43 ± 1.76
NLMR	1.47 ± 0.96
SBP (mmHg)	127.61 ± 16.79
DBP (mmHg)	71.97 ± 12.37
MAP (mmHg)	90.52 ± 12.09
PP (mmHg)	55.63 ± 14.97
Ca(mg/dL)	9.12 ± 0.52
P (mg/dL)	3.91 ± 0.92
CaxP product (mg^2^/dL^2^)	35.75 ± 12.65
PTH (pg/mL)	104.45 ± 106.56
Uric acid (mg/dL)	5.59 ± 1.83
LDLcholesterol (mg/dL)	83.15 ± 28.75
*hs*-CRP (mg/dL)	0.23 ± 0.11
ABI	1.08 ± 0.14

Data are expressed as mean ± SD or n (%).

NDD-CKD: non-dialysis-dependent chronic kidney disease; DM: diabetes mellitus; HTN: hypertension; eGFR: estimated glomerular filtration rate; CKD-EPI: CKD-Epidemiology Collaboration; NTR: neutrophil-to-total leukocyte count ratio; LTR: lymphocyte-to-total leukocyte count ratio; MTR: monocyte-to-total leukocyte count ratio; NLR: neutrophil-to-lymphocyte count ratio; LMR: lymphocyte-to-monocyte count ratio; NMR: neutrophil-to-monocyte count ratio; NLMR: neutrophil-to-sum of lymphocyte and monocyte count ratio.

### Comparisons of variables by eGFR level

Total leukocyte, neutrophil count (/mm^3^), NLR, and NLMR in patients with an eGFR 60–89 mL/min/1.73 m^2^ and eGFR < 60 mL/min/1.73 m^2^ (5871.43 ± 1518.60, 3340.48 ± 1336.72, 1.75 ± 0.71, 1.75 ± 0.71; and 6773.68 ± 1818.71, 3771.58 ± 1345.50, 1.72 ± 0.75, 1.35 ± 0.49, respectively; *p* < 0.05) were significantly higher than those in healthy controls (4809.09 ± 1278.64, 2462.73 ± 817.83, 1.31 ± 0.33, and 1.09 ± 0.54, respectively). Lymphocyte and monocyte count (/mm^3^), and ABI in patients with an eGFR < 60 mL/min/1.73 m^2^ (2322.17 ± 798.87, 557.72 ± 203.54, and 1.07 ± 0.15, respectively; *p* < 0.05) were higher than those in healthy controls and patients with an eGFR 60–89 mL/min/1.73 m^2^). Other comparisons of variables by eGFR level are shown in Table 2.

**Table 2.** Comparisons of variables by eGFR level (*N* = 100).

	Control (*n* = 21)	eGFR 60–89 mL/min/1.73 m^2^ (*n* = 21)	eGF*R* < 60 mL/min/1.73 m^2^ (*n* = 58)
Age (years)	63.91 ± 3.56	61.19 ± 10.58	63.43 ± 12.62
Male:female	11:10	11:10	41:17**
DM:non-DM		3:18	19:39
Total leukocyte count (/mm^3^)	4809.09 ± 1278.64	5871.43 ± 1518.60*	6773.68 ± 1818.71*
Neutrophil count (/mm^3^)	2462.73 ± 817.83	3340.48 ± 1336.72*	3771.58 ± 1345.50*
Lymphocyte count (/mm^3^)	1875.82 ± 403.82	1951.57 ± 387.82	2322.17 ± 798.87**
Monocyte count (/mm^3^)	371.82 ± 158.80	476.67 ± 138.43	557.72 ± 203.54**
NTR	0.50 ± 0.06	0.55 ± 0.09	0.55 ± 0.09
LTR	0.40 ± 0.06	0.34 ± 0.07*	0.35 ± 0.08*
MTR	0.08 ± 0.02	0.08 ± 0.02	0.08 ± 0.02
NLR	1.31 ± 0.33	1.75 ± 0.71*	1.72 ± 0.75*
NMR	6.84 ± 1.44	7.23 ± 2.51	7.25 ± 2.55
LMR	5.51 ± 1.77	4.31 ± 1.17	4.60 ± 1.83
NLMR	1.09 ± 0.54	1.75 ± 0.71*	1.35 ± 0.49*
SBP (mmHg)	129.82 ± 10.97	124.10 ± 13.07	128.88 ± 17.88
DBP (mmHg)	81.64 ± 9.34	73.33 ± 8.03*	71.48 ± 13.63*
MAP (mmHg)	97.70 ± 9.50	90.25 ± 9.13*	91.44 ± 11.54*
PP (mmHg)	48.18 ± 6.00	50.76 ± 8.61	56.18 ± 13.62**
Ca(mg/dL)	9.11 ± 0.20	9.16 ± 0.33	9.11 ± 0.57
P (mg/dL)	3.71 ± 0.53	3.45 ± 0.46	4.07 ± 1.00***
CaxP product (mg^2^/dL^2^)	33.79 ± 4.84	31.64 ± 4.47	36.98 ± 8.75***
PTH (pg/mL)			104.45 ± 106.56
Uric acid (mg/dL)	4.85 ± 1.04	4.61 ± 1.25	5.94 ± 1.88**
LDLcholesterol (mg/dL)	81.36 ± 27.95	87.48 ± 33.11	81.56 ± 27.11
*hs*-CRP (mg/dL)	0.11 ± 0.16	0.13 ± 0.23	0.11 ± 0.13
ABI	1.12 ± 0.06	1.12 ± 0.06	1.07 ± 0.15**

*p < 0.05 vs control, **p < 0.05 vs control, eGFR 60–89 mL/min/1.73 m^2^, ***p < 0.05 vs eGFR 60–89 mL/min/1.73 m^2^.

ABI: ankle-brachial index; NDD-CKD: non-dialysis-dependent chronic kidney disease; NTR: neutrophil-to-total leukocyte count ratio; LTR: lymphocyte-to-total leukocyte count ratio; MTR: monocyte-to-total leukocyte count ratio; NLR: neutrophil-to-lymphocyte count ratio; LMR: lymphocyte-to-monocyte count ratio; NMR: neutrophil-to-monocyte count ratio; NLMR: neutrophil-to-sum of lymphocyte and monocyte count ratio.

### Comparisons of variables by median ABI in NDD-CKD patients

We excluded patients with DD-CKD, PAOD such as gangrenous changes in both foot, amputees, and patients undergoing PTA and CLI. And only 5 patients had an ABI value <0.90 and 6 patients showed an ABI value <1.00 in our study. Therefore, we decided to compare selected clinical, anthropometric, biochemical variables and leukocyte subtypes using median value of ABI although it is not line with the most current international guidelines for the evaluation of PAOD (high risk <0.90; intermediate risk 0.90–1.00) [17]. The median value of ABI was 1.10 in our study. The mean monocyte (/mm^3^) count and MTR in the ABI value <1.10 group were significantly higher than those in the ABI value ≥1.10 group (573.78 ± 189.64 and 0.089 ± 0.022 vs 484.75 ± 154.99 and 0.078 ± 0.021, respectively; *p* < 0.05) (Table 3). There were no significant differences in total leukocyte, neutrophil, or lymphocyte count and NTR, LTR, NLR, NMR, LMR, and NLMR between the ABI value ≥1.10 and ABI value <1.10 groups (Table 3).

**Table 3.** Comparisons of variables by median ABI in NDD-CKD patients (*N* = 79).

	ABI ≥ 1.10 (*n* = 41)	ABI < 1.10 (*n* = 38)
Age (years)	61.83 ± 11.93	63.92 ± 12.31
Male:female	17:24	10:28
DM:non-DM	10:31	12:26
eGFR (mL/min/1.73m^2^)	36.23 ± 25.18	34.65 ± 22.81
Total leukocyte count (/mm^3^)	6267.50 ± 1575.35	6662.16 ± 1759.95
Neutrophil count (/mm^3^)	3557.00 ± 1313.67	3625.14 ± 1137.67
Lymphocyte count (/mm^3^)	2114.72 ± 557.67	2340.32 ± 877.92
Monocyte count (/mm^3^)	**484.75 ± 154.99**	573.78 ± 189.64*
NTR	0.56 ± 0.09	0.54 ± 0.08
LTR	0.35 ± 0.08	0.35 ± 0.08
MTR	**0.078 ± 0.021**	0.089 ± 0.022*
NLR	1.67 ± 0.61	1.64 ± 0.59
NMR	7.73 ± 2.68	6.72 ± 2.28
LMR	4.75 ± 1.85	4.28 ± 1.44
NLMR	1.41 ± 0.55	1.31 ± 0.44
SBP (mmHg)	130.64 ± 14.86	124.50 ± 18.71
DBP (mmHg)	74.36 ± 9.23	70.98 ± 9.37
MAP (mmHg)	93.12 ± 10.12	88.83 ± 11.51
PP (mmHg)	56.28 ± 11.25	53.51 ± 13.87
Ca(mg/dL)	9.11 ± 0.47	9.13 ± 0.57
P (mg/dL)	3.90 ± 0.71	3.92 ± 1.12
CaxP product	35.49 ± 6.44	35.65 ± 9.78
PTH (pg/mL)	110.40 ± 112.67	99.09 ± 102.39
Uric acid (mg/dL)	5.49 ± 1.71	5.69 ± 1.96
LDL cholesterol (mg/dL)	79.49 ± 29.52	87.22 ± 27.69
*hs*-CRP (mg/dL)	0.13 ± 0.23	0.11 ± 0.13

The bold values represents as Statistically significant *p < 0.05 vs ABI ≥ 1.10. ABI: ankle-brachial index; NDD-CKD: non-dialysis dependent chronic kidney disease; NTR: Neutrophil-to-total leukocyte count ratio; LTR: lymphocyte-to-total leukocyte count ratio; MTR: monocyte-to-total leukocyte count ratio; NLR: neutrophil-to-lymphocyte count ratio; LMR: lymphocyte-to-monocyte count ratio; NMR: neutrophil-to-monocyte count ratio; NLMR: neutrophil-to-sum of lymphocyte and monocyte count ratio.

### Correlation analysis between variables and ABI in NDD-CKD patients

**Figure 1.**
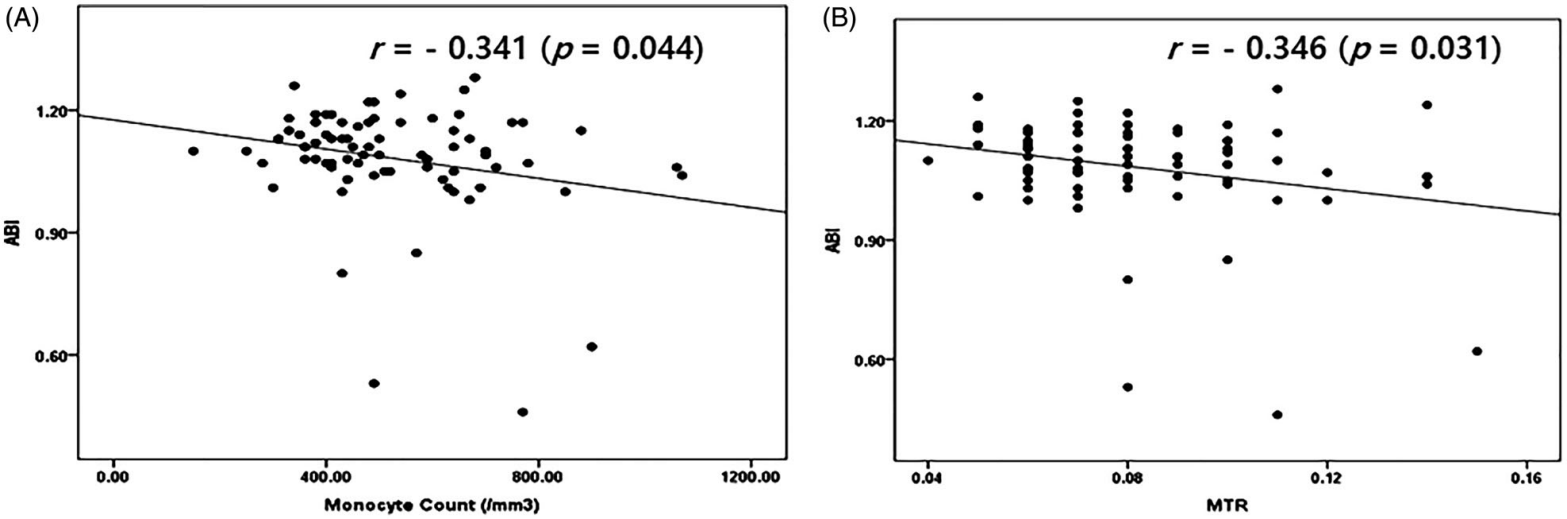
(A, B) Correlations between monocyte count, MTR, and ABI in NDD-CKD patients (*N* = 79).

ABI values showed a negative correlation with monocyte count (*r*= −0.341, *p* = 0.044) and MTR (*r*= −0.346, *p* = 0.031) (Figure 1(A,B)).

### Multivariate linear regression analysis with ABI as a dependent variable in NDD-CKD patients

Multivariate linear regression analyses showed that monocyte count were negatively associated with ABI values (*β ± SE* = −1.825 ± 0.341, *p* = 0.013) (Table 4). However, we were unable to demonstrate the association between other variables including age, sex, presence of DM, SBP, DBP, MAP, PP, CaxP product, uric acid, LDL-cholesterol, *hs*-CRP, total leukocyte, neutrophil and lymphocyte count or NLR, NMR, LMR, NLMR and ABI values.

**Table 4.** Multivariate linear regression analysis with ABI values as a dependent variable in NDD-CKD patients (*N* = 79).

	ABI		
	*β*	*SE*	*p* Value
**Monocyte count**	−1.825	0.341	0.013
**Age**	−0.234	0.001	0.047
Sex	0.060	0.032	0.617
Presence of DM	−0.032	0.032	0.658
SBP	0.358	0.001	0.709
DBP	−0.048	0.001	0.718
MAP	−0.118	0.003	0.685
PP	−0.147	0.003	0.573
Ca x P product	−0.040	0.017	0.730
Uric acid	−0.040	0.008	0.719
LDL-cholesterol	−0.226	0.001	0.055
hs-CRP	0.154	0.031	0.291
Total leukocyte count	0.315	0.019	0.515
Neutrophil count	0.406	0.023	0.406
Lymphocyte count	0.096	0.009	0.754
NLR	0.046	0.021	0.920
NMR	−0.239	0.021	0.532
LMR	−0.118	0.021	0.690
NLMR	−0.047	0.089	0.941

Selected variables: age, sex, presence of DM, SBP, DBP, MAP, PP, CaxP product, uric acid, LDL-cholesterol, hs-CRP, total leukocyte, neutrophil, monocyte and lymphocyte count or NLR, NMR, LMR, and NLMR.

NLR: neutrophil-to-lymphocyte count ratio; LMR: lymphocyte-to-monocyte count ratio; NMR: neutrophil-to-monocyte count ratio; NLMR: neutrophil-to-sum of lymphocyte and monocyte count ratio.

### Receiver operating characteristics (ROC) curves of monocyte counts for predicting an ABI value <1.10 in NDD-CKD patients

**Figure 2.**
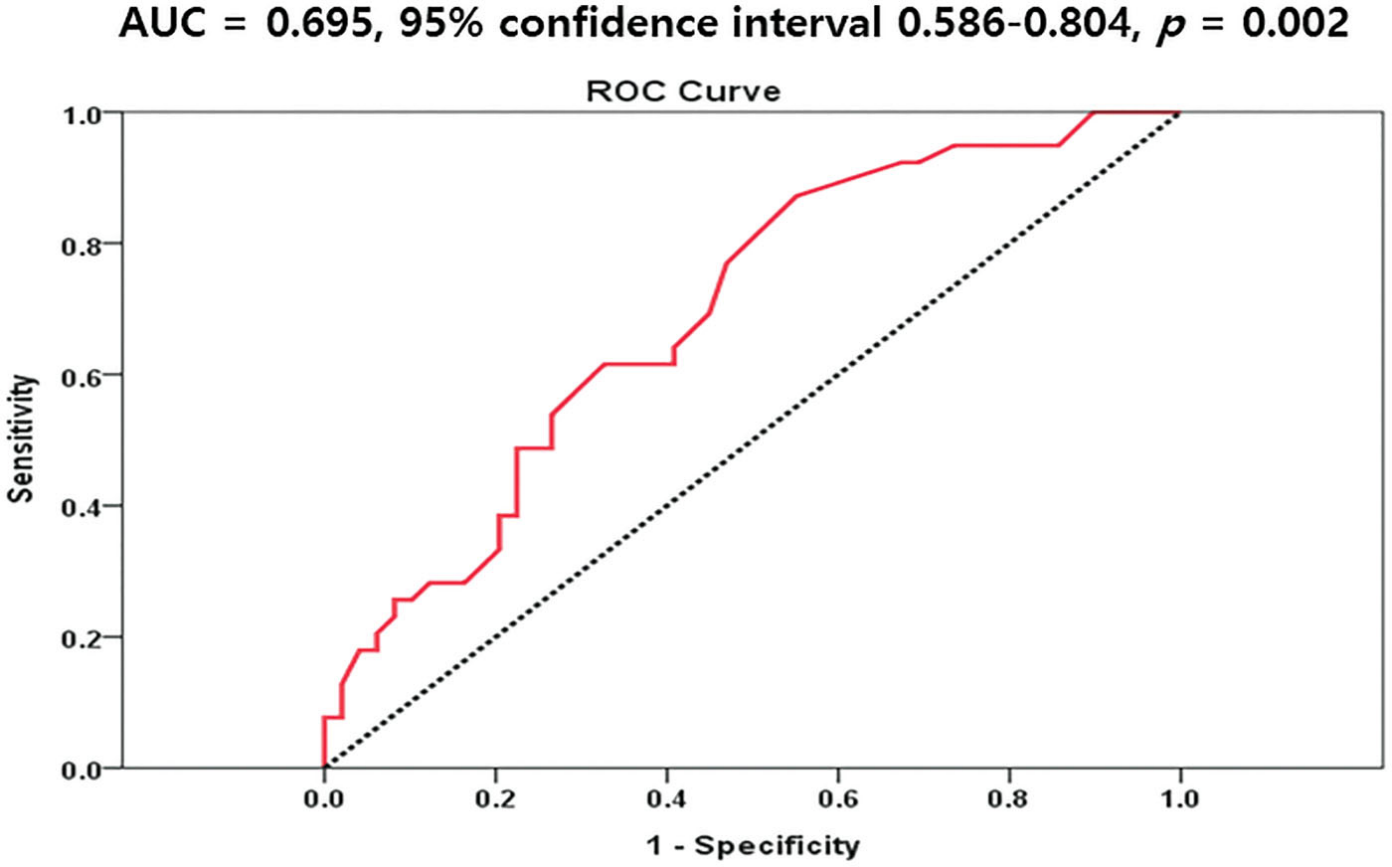
Receiver operating characteristics (ROC) curves of monocyte counts for predicting ABI < 1.10.

We evaluated the ROC curve of monocyte counts for predicting ABI <1.10. The area under the curve of monocyte counts was 0.695 (95% confidence interval 0.586–0.804, *p* = 0.002) in predicting an ABI value <1.10 (Figure 2). Optimal cutoff value of monocyte counts was 575/mm^3^ (sensitivity 62.5%, specificity 72.0%).

## Discussion

This cross-sectional study demonstrated that mean ABI in patients with an eGFR < 60 mL/min/1.73 m^2^ was significantly lower than that in healthy controls and patients with an eGFR 60–89 mL/min/1.73 m^2^. Our results showed that monocyte counts and MTR in patients with an ABI value <1.10 were significantly higher than those in patients with an ABI value ≥1.10. In particular, monocyte counts were negatively correlated and associated with ABI values and could be a predictor for an ABI value <1.10 in patients with NDD-CKD who did not suffer from apparent PAOD and CLI. This relationship was not present for other leukocyte subtypes despite total leukocyte, neutrophil, and lymphocyte counts being significantly increased in NDD-CKD patients compared to normal controls.

Leukocytes are a strong independent risk factor for cardiovascular events [18] and the prevalence and progression of sub-clinical atherosclerosis [19]. A recent meta-analysis concluded that, among leukocyte types, neutrophils were more strongly associated with future coronary events than monocytes [20]. Recently, Ito *et al* published that renal function was a strong determinant of NLR in cardiovascular outpatients. NLR elevation due to renal impairment is caused by distortion of the hematopoietic process in bone marrow [21]. Gary T *et al* demonstrated that an increased NLR was significantly associated with patients at high risk for CLI, PAOD, prior myocardial infarction (MI) and stroke [22]. The NLR was an easy to perform test, which could be used to emphasize patients at high risk for above disorders [22]. The NLR is derived from the value of neutrophils and lymphocytes, two different types of leukocytes. Neutrophils secrete inflammatory mediators that can lead to vascular wall degeneration [23]. Conversely, lymphocytes regulate the inflammatory response and have an antiatherosclerotic role in which regulatory T-cell, a subclass of lymphocyte, may have an inhibitory effect on atherosclerosis [23]. Iso et al. investigated which factors influence limb salvage after bone marrow mononuclear cell implantation (BMI) in patients with chronic CLI [24]. They demonstrated that the cells implanted in the limb salvage group were composed of significantly higher numbers of lymphocytes [24]. And they suggested that lymphocytes played an important role in patients with chronic CLI after BMI [24]. Stabile et al. described the above findings as follows; CD8+ T lymphocytes regulated the arteriogenic response to ischemia by infiltrating the site of collateral vessel development and recruiting CD4+ mononuclear cells through the expression of interleukin-16 in mouse model of unilateral hindlimb ischemia [25]. Therefore, as in patients with a high lymphocyte count, leading to a lower NLR might have more collateral growth leading to less ischemia and less CLI. In addition, Turak et al. stressed that high preprocedural NLR was a powerful and independent predictor of bare-metal stent restenosis in patients with stable and unstable angina pectoris [26]. The authors demonstrated that an NLR > 2.73 had 80% sensitivity and 75% specificity for predicting stent restenosis. The NLR seems to reflect the active atherosclerotic disease in these patients, which leads to an elevated risk for restenosis after bare metal stent insertion [26]. However, our results showed no significant association between neutrophil, lymphocyte, NLR, and ABI values in NDD-CKD patients. The main reason of this discrepancy between this and previous studies may be related to differences in enrolled patients. That is, our study enrolled patients with no apparent PAOD and CLI, whereas previous studies enrolled patients with PAOD and CLI. In particular, the cutoff value of NLR level (>3.95) suggested by Gary et al. [22] was much higher than that in our study.

Activation of monocytes and differentiation into lipid-laden macrophages are fundamental events in the formation of atherosclerotic lesions [27]. Marginalization of monocytes along the endothelium and transmigration into the intimal spaces appears to depend on the local presence of high amounts of oxidized low-density lipoprotein cholesterol and are mediated by adhesion molecules [27]. Monocyte count in blood was found to be a better cross-sectional marker of plaque presence than IL-6, high-sensitive C-reactive protein, fibrinogen, and white blood cells [28]. In PAOD patients, monocytes also seem to play an important role as reported by Dopheide *et al.* They were able to show that PAOD patients had a significantly higher proportion of pro-inflammatory monocytes than healthy individuals [29]. Nasir et al. published that monocytes were the only leukocyte type significantly and independently associated with PAOD in a representative sample of the US population after adjustment for other inflammatory markers [30]. However, the above studies differed from ours in that they targeted patients who already had PAOD. The LMR is derived from the value of lymphocytes and monocytes, two different parts of the leukocytes. A paper suggested that as patients with a low lymphocyte and high monocyte count, leading to a low LMR, might have less collateral growth, in turn leading to more ischemia and therefore more CLI [11]. In our results, monocyte counts in patients with an eGFR < 60 mL/min/1.73 m^2^ were significantly higher than controls and in patients with an eGFR 60–89 mL/min/1.73 m^2^ and monocyte counts, and the MTR in patients with an ABI value <1.10 were significantly higher than those in patients with an ABI value ≥1.10. In addition, monocyte counts and MTR were negatively correlated with and/or affected ABI values in NDD-CKD patients who do not suffer from apparent PAOD/CLI, although there was no correlation between LMR and ABI. Taken together, our data suggested that monocytes only among leukocyte subtypes are negatively associated with ABI values in NDD-CKD patients who do not suffer from apparent PAOD and CLI. CKD is characterized by systemic inflammation and disturbances in the blood leukocytes that remain incompletely understood. In particular, abnormalities in the numbers and relative proportions of the three major monocyte subsets—classical, intermediate, and non-classical—are described in CKD and end-stage renal disease [31]. Naicker et al. confirmed monocyte subset dysregulation in CKD and identified a distinct subpopulation of intermediate monocytes that was associated with higher rate of loss of kidney function [32]. So, it would be interesting to determine which monocyte subsets (classical/intermediate/nonclassical) are increased in individuals with CKD who have lower ABI values.

This study has some limitations. First, the number of patients was relatively small, single-center and cross-sectional study without follow-up data. Therefore, correlation coefficients in our study were quite weak. DM, a traditional cardiovascular risk factor, can affect ABI value. However, there was no significant relation between DM and ABI values in our study. It is because we excluded patients with DD-CKD, PAOD such as gangrenous changes in both foot, amputees, and patients undergoing PTA and CLI. And only 5 patients had an ABI value <0.90 and 6 patients showed an ABI value <1.00 in our study. Nevertheless, since the statistical significance was clearly identified in our study, we thought above problem could be overcome by increasing the number of enrolled patients. Second, the number of monocytes in peripheral smear is very reactive and variable number and depends on the imminent status of the patients. Factors such as infections, day-to-day variations, and variability in leukocyte determination may have affected our results. However, we enrolled patients having the results of CBC more than three times a year prior to the ABI measurements except controls in order to overcome above limitations and the average value of three CBC tests was used in our study. In addition, *hs*-CRP was within the normal range in our study population.

## Conclusion

To our knowledge, it is true that there have been no studies to investigate the relationship between ABI values and total/partial leukocyte counts or subtypes, especially in NDD-CKD patients with no evidences of PAOD and CLI. Our data demonstrated that monocyte counts and MTR were negatively correlated with ABI values and that monocyte counts were negatively associated with ABI values in patients with NDD-CKD who do not suffer from apparent PAOD and CLI. This relationship was not present for other leukocyte types, although total leukocyte, neutrophil, and lymphocyte counts were significantly increased in NDD-CKD patients versus normal controls. Therefore, it is thought that periodic monitoring of monocyte counts would be necessary in patients with NDD-CKD. Its role in the possible prevention of the progression of PAOD in patients with NDD-CKD should be elucidated in the near future.
